# SOX Transcription Factors as Important Regulators of Neuronal and Glial Differentiation During Nervous System Development and Adult Neurogenesis

**DOI:** 10.3389/fnmol.2021.654031

**Published:** 2021-03-31

**Authors:** Milena Stevanovic, Danijela Drakulic, Andrijana Lazic, Danijela Stanisavljevic Ninkovic, Marija Schwirtlich, Marija Mojsin

**Affiliations:** ^1^Laboratory for Human Molecular Genetics, Institute of Molecular Genetics and Genetic Engineering, University of Belgrade, Belgrade, Serbia; ^2^Faculty of Biology, University of Belgrade, Belgrade, Serbia; ^3^Serbian Academy of Sciences and Arts, Belgrade, Serbia

**Keywords:** SOX transcription factors, neuronal differentiation, glial differentiation, adult neurogenesis, signaling pathways, microRNA

## Abstract

The SOX proteins belong to the superfamily of transcription factors (TFs) that display properties of both classical TFs and architectural components of chromatin. Since the cloning of the *Sox*/*SOX* genes, remarkable progress has been made in illuminating their roles as key players in the regulation of multiple developmental and physiological processes. SOX TFs govern diverse cellular processes during development, such as maintaining the pluripotency of stem cells, cell proliferation, cell fate decisions/germ layer formation as well as terminal cell differentiation into tissues and organs. However, their roles are not limited to development since SOX proteins influence survival, regeneration, cell death and control homeostasis in adult tissues. This review summarized current knowledge of the roles of SOX proteins in control of central nervous system development. Some SOX TFs suspend neural progenitors in proliferative, stem-like state and prevent their differentiation. SOX proteins function as pioneer factors that occupy silenced target genes and keep them in a poised state for activation at subsequent stages of differentiation. At appropriate stage of development, SOX members that maintain stemness are down-regulated in cells that are competent to differentiate, while other SOX members take over their functions and govern the process of differentiation. Distinct SOX members determine down-stream processes of neuronal and glial differentiation. Thus, sequentially acting SOX TFs orchestrate neural lineage development defining neuronal and glial phenotypes. In line with their crucial roles in the nervous system development, deregulation of specific SOX proteins activities is associated with neurodevelopmental disorders (NDDs). The overview of the current knowledge about the link between *SOX* gene variants and NDDs is presented. We outline the roles of SOX TFs in adult neurogenesis and brain homeostasis and discuss whether impaired adult neurogenesis, detected in neurodegenerative diseases, could be associated with deregulation of SOX proteins activities. We present the current data regarding the interaction between SOX proteins and signaling pathways and microRNAs that play roles in nervous system development. Finally, future research directions that will improve the knowledge about distinct and various roles of SOX TFs in health and diseases are presented and discussed.

## Introduction

The development of multicellular organisms and the maintenance of homeostasis in adulthood are achieved by complex control of basic cellular processes such as the maintenance of pluripotent stem cells, cell fate decision, differentiation, proliferation, and cell death. One of the key mechanisms involved in the control of developmental processes is based on the transcriptional regulation of gene expression. Through the activation and repression of the target genes, transcription factors (TFs) determine the fate of cells within tissues, organs and organisms, controlling the development. Most TFs act within complex regulatory networks, enabling combinatorial regulation of gene expression within the cells. Numerous families of genes encoding TFs involved in the control of embryonic development have been discovered, including the *SOX* gene family.

## SOX Transcription Factors

*Sry* (Sex-determining Region Y), a founder member of the *Sox* gene family, was discovered in 1990 as a sex-determining gene necessary and sufficient to specify the male phenotype (Gubbay et al., 1990; Sinclair et al., 1990). During the course of cloning of *Sry*, the presence of related genes was discovered sharing the homology with the HMG box of *Sry*. These newly identified genes have been named by the acronym *Sox*/*SOX* (in mammals and human, respectively) standing for *Sry*-related HMG box genes (Denny et al., 1992; Wright et al., 1993). Further, it was shown that the *SOX* family is multigenic, with new members discovered both in vertebrates and invertebrates and being assigned by numbers based on the order of their discovery. After a detailed insight, the presence of 20 *SOX* genes in human genome was identified (Table 1) providing the basis for their final re-numeration and classification (Schepers et al., 2002). Further research has shown that *SOX* genes encode the family of diverse and well conserved TFs.

**TABLE 1:** Classification of the human SOX genes.

Group	Gene		Gene locus	References
*SOXA*	*SRY*		Yp11.3	Sinclair et al., 1990
*SOXB*	*SOXB1*	*SOX1*	13q34	Malas et al., 1997
		*SOX2*	3q26.3	Stevanovic et al., 1994
		*SOX3*	Xq26.3	Stevanovic et al., 1993
	*SOXB2*	*SOX14*	3q23	Arsic et al., 1998; Malas et al., 1999
		*SOX21*	13q31-q32	Malas et al., 1999
*SOXC*	*SOX4*		6p22.3	Farr et al., 1993
	*SOX11*		2p25	Jay et al., 1995
	*SOX12*		20p13	Jay et al., 1997
	(*SOX22 is renamed as SOX12*)			
*SOXD*	*SOX5*		12p12.1	Wunderle et al., 1996
	*SOX6*		11p15.3	Cohen-Barak et al., 2001
	*SOX13*		1q32	Argentaro et al., 2000
*SOXE*	*SOX8*		16p13.3	Pfeifer et al., 2000
	*SOX9*		17q23	Foster et al., 1994
	*SOX10*		22q13	Pusch et al., 1998
*SOXF*	*SOX7*		8p22	Takash et al., 2001
	*SOX17*		8q11.23	Katoh, 2002
	*SOX18*		20q13.33	Stanojcic and Stevanovic, 2000
*SOXG*	*SOX 15*		17p13	Meyer et al., 1996;
	*(SOX20 is renamed as SOX15)*			Vujic et al., 1998
*SOXH*	*SOX30*		5q33	Osaki et al., 1999

*Sox12 and SOX22, as well as, Sox15 and SOX20 denominate the same SOX proteins in mouse and human, respectively.*

Based on the structure, expression profiles, as well as the similarity between the proteins they encode, human *SOX* genes are divided into 8 groups, A to H (Table 1), with group B being further split into subgroups B1 (*SOX1*, *SOX2*, and *SOX3*) and B2 (*SOX14* and *SOX21*) (Uchikawa et al., 1999). SOX proteins within the same group show a high level of homology, both within and outside the HMG domain, while proteins from different groups show homology only within the HMG domain (Bowles et al., 2000). Apart from the genes *SRY* and *SOX3*, other family members are located on autosomes and scattered throughout the genome (Table 1). Majority of *SOX* family members are single exon genes, with the exception of genes *SOX5*, *SOX9*, *SOX10*, *SOX15*, *SOX17*, and *SOX18* that possess multiple exons.

The SOX proteins display properties of both classical TFs and architectural components of chromatin (reviewed in Pevny and Lovell-Badge, 1997). SOX proteins carry an HMG domain of 79 amino acids that enables their specific binding to the sequence (A/T A/T CAA A/T) (Harley et al., 1994) and additional domains involved in transcriptional regulation (reviewed in Pevny and Lovell-Badge, 1997). In contrast to the majority of DNA-binding proteins, SOX proteins interact with the minor groove and, upon binding, they introduce strong bends into DNA (reviewed in Wegner, 1999). Consequently, SOX proteins act as architectural proteins by shaping the gene regulatory regions and by enabling establishment of physical contacts between TFs bound on the same target gene promoter or enhancer (reviewed in Wegner, 2005). SOX TFs exert regulatory functions by activating or repressing gene transcription only through specific interactions with a partner factor(s) and by establishing contacts with the basic transcription machinery (Kamachi et al., 2000).

The SOX TFs perform unique functions in different cell types and regulate different events in the same cell type. Several SOX proteins are demonstrated to have the ability to pair off with various types of TFs (Kamachi et al., 2000) and their specificity is achieved via binding partners (reviewed in Bernard and Harley, 2010). Consequently, transcriptional regulatory functions of SOX proteins usually require the cooperation with interacting partner factors that bind DNA in the vicinity of the SOX site and allow specific selection of target genes (reviewed in Kamachi et al., 2000; Kondoh and Kamachi, 2010). SOX partner factor cooperation is dynamic and changes in partner factors enable SOX proteins to regulate different events in the same cell type and to drive the progression of developmental processes (reviewed in Kondoh and Kamachi, 2010).

Since discovery, essential roles have been assigned to SOX TFs. Their critical functions have been revealed by both studying naturally occurring mutations in humans, as well as, by targeted mutations introduced in animal models. Numerous studies aimed at discovery of the roles of *Sox*/*SOX* genes are often being complicated by pleiotropy and by partial or extensive functional redundancy among co-expressed members of the same groups (Kamachi and Kondoh, 2013).

It has been shown that many developmental processes depend on the presence of SOX proteins, ranging from blastocyst formation, gastrulation, germ layer formation to development of adult tissues and organs (reviewed in Wegner, 2005; Lefebvre et al., 2007; Kamachi and Kondoh, 2013). SOX TFs have been implicated in preimplantation development. Expression of *Sox2* was detected from oocyte, through 2-cell to 8-cells embryo and morula to the blastocyst (Keramari et al., 2010). Study of the effects of *Sox2* knockdown in preimplantation embryo suggested that first essential function of *Sox2* is to facilitate establishment of the trophectoderm lineage (Keramari et al., 2010). Expression of *Sox2* is detected in the inner cell mass of the murine blastocyst and subsequently in primitive ectoderm, extraembryonic ectoderm (Avilion et al., 2003) and the developing nervous system (Collignon et al., 1996).

The SOX TFs govern diverse cellular processes during development, such as maintaining the pluripotency of stem cells, cell proliferation, cell fate decisions, germ layer formation as well as terminal cell differentiation into tissues and organs (reviewed in Reiprich and Wegner, 2015; She and Yang, 2015). However, their roles are not limited to development since SOX TFs influence survival, regeneration, cell death and control homeostasis in adult tissues (Pevny and Placzek, 2005; Mercurio et al., 2019). As reported in numerous publications, most cells express at least one *Sox/SOX* gene and various members of the *SOX* gene family have roles in many tissues and stages of development (reviewed in Pevny and Lovell-Badge, 1997; Wegner, 1999; Kiefer, 2007; Kamachi and Kondoh, 2013). For instance, tissues that require *Sox2* during development continue to express this factor in some adult stem and progenitor cells derived from that tissue. Thus, *Sox2* marks stem and progenitor cell populations in adult tissues that depend on *Sox2* expression during development (Sarkar and Hochedlinger, 2013).

## SOX Proteins and Pluripotency

A unique set of TFs is required to establish embryonic stem cells (ESCs) and to maintain their pluripotent and proliferative state. The numerous evidences reveal the roles of SOX proteins in preservation of stem cell characteristics.

**FIGURE 1:**
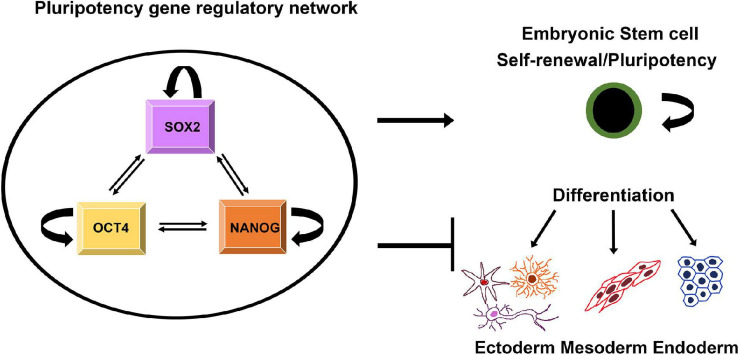
Schematic illustration of regulatory network that controls pluripotency, self-renewal and differentiation. SOX2, NANOG, and OCT4 regulate their own expression targeting both their own promoters and those of each other. The triad contributes to maintaining pluripotency of ESCs by activating genes involved in pluripotency and by repressing genes linked to lineage commitment (Scheper and Copray, 2009).

As mentioned above, SOX proteins have the ability to pair off with various types of TFs and regulatory functions of SOX proteins usually require the cooperation with interacting partner factors (Kamachi et al., 2000). SOX2, together with OCT4 (octamer-binding transcription factor 4) and NANOG (named as abbreviation for the mythological Celtic land of the ever-young, “Tir nan Og”) (Cavaleri and Scholer, 2003), establish the core transcriptional circuit that orchestrate self-renewal and maintenance of pluripotency of the stem cells (Figure 1) (Rodda et al., 2005).

This pluripotency gene regulatory network relies on direct physical interaction between SOX2, NANOG, and OCT4 (Ambrosetti et al., 2000; Gagliardi et al., 2013). Through a cooperative interaction these factors drive pluripotent-specific expression of the numerous genes and play key roles in determining the fate of ESCs, regulating two distinct and opposing functions: self-renewal and differentiation (Figure 1) (Rizzino, 2008). Besides directing the expression of target genes, SOX2, OCT4, and NANOG regulate their own expression via positive-feedback loops (Figure 1) (Boyer et al., 2005). In addition, this fully-connected triad has been implicated as recurring network motif among the transcriptional regulatory circuits that control the development and maintenance of cellular states (Faucon et al., 2014). In addition to SOX2, SOX15 is expressed in mouse ESCs and associated with Oct3/4 (Maruyama et al., 2005). It was found that SOX15 is able to replace the function of SOX2 in self-renewal of mouse ESCs (Niwa et al., 2016).

Since SOX2 is a part of integrated and self-controlling network, the level of its expression is critical to sustain the stemness phenotype. Accordingly, SOX2 overexpression reduced the level of OCT4 and NANOG in human ESCs (Adachi et al., 2010). In line with this data, we detected downregulation of *OCT4* gene expression in pluripotent embryonal carcinoma stem cells NT2/D1 with constitutive SOX2 overexpression (Drakulic et al., 2012). We also demonstrate that transition from proliferation to retinoic acid (RA) induced neural differentiation of NT2/D1 cells coincides with complete OCT4 down-regulation (Stevanović et al., 2017). However, SOX2 overexpressing NT2/D1 cells retain ability to differentiate (Drakulic et al., 2012; Klajn et al., 2014) even in the presence of elevated *SOX2* expression after 21 days of treatment with RA (Drakulic et al., 2012).

In ESCs, SOX2 overexpression rapidly induces the expression of another SOXB member – SOX21 that further influences fate of these cells (Mallanna et al., 2010). Subsequently, SOX21 acting as a repressor, disrupts ESCs self-renewal and induces differentiation (Mallanna et al., 2010).

In addition, SOX2 plays important role in reprogramming adult cells and generation of induced pluripotent stem cells (iPSCs). Reprogramming is achieved by overexpression of stem cell-associated genes in differentiated cells, such as adult fibroblasts (Takahashi et al., 2007; Wernig et al., 2007; Takahashi and Yamanaka, 2016). SOX2 is recognized as one of the “magical four” crucial TFs capable of cooperating to reprogram differentiated cells into an iPSCs (Qi and Pei, 2007). The fact that SOX2 is crucial factor for reversing the somatic cells back to their pluripotent state (Takahashi and Yamanaka, 2016) demonstrates its pivotal role in maintenance of cell pluripotency.

Apart from *Sox2*, other members of the *Sox* gene family may also be involved in the reprogramming process. *Sox1* yields iPSCs with a similar efficiency as *Sox2*, while *Sox3*, *Sox*15, and *Sox18* genes are also capable to generate iPSCs, although with decreased efficiency (Nakagawa et al., 2008).

## SOX Proteins as Pioneer Factors

The SOX proteins also function as pioneer factors that occupy silenced target genes and keep them in a poised state for activation at subsequent stages of differentiation (Bergsland et al., 2011; Zaret and Carroll, 2011).

**FIGURE 2:**
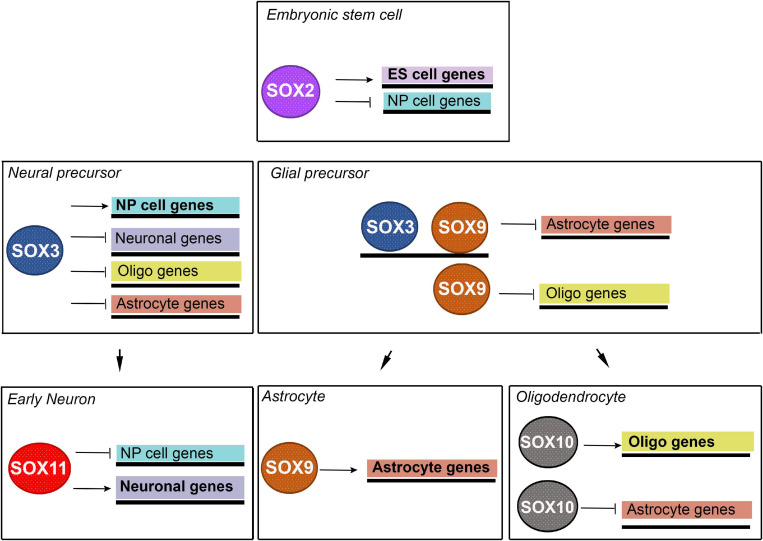
Schematic illustration of proposed model for neuronal and glial lineage specification governed by sequentially acting SOX TFs. Neural precursor cell specific genes are repressed in ESCs by SOX2 and activated in neural precursor cells by SOX3. Neuronal specific genes are repressed in neural precursor cells by SOX3 and activated in early neurons by SOX11. Astrocyte specific genes are repressed in neural precursor cells by SOX3, in glial precursors by SOX3 and SOX9 and in oligodendrocyte by SOX10. These genes are activated in astrocytes by SOX9. Oligodendrocyte specific genes are repressed in neural precursor cells by SOX3, in glial precursor cells by SOX9 and activated in oligodendrocytes by SOX10. NP- neural precursors; Oligo – oligodendrocyte. [Modified from Bergsland et al. (2011) and Klum et al. (2018)].

Bergsland et al. (2011) demonstrated that binding of SOX proteins is developmental stage-specific and revealed sequential binding of SOX proteins to a common set of neural genes. Prebinding of SOX proteins to silent genes facilitates those genes to be activated at later stages of neural development. They showed that expression of many genes that are targeted by binding of SOX2 in ESCs and neural precursors is first initiated in neural precursors, while many neuronal genes that are prebound by SOX2 and SOX3 in neural precursors can only be activated by SOX11 in differentiating neurons (Figure 2) (Bergsland et al., 2011). By these data, the authors reveal that sequentially acting SOX TFs coordinate neural gene expression from pluripotent cells to later stages of neuronal development.

It has been demonstrated that glial-specific gene sets are extensively preselected in multipotent neural progenitor cells (NPCs) through prebinding by SOX3 (Klum et al., 2018). Further, in the subsequent lineage-restricted glial precursor cells, it was shown that SOX3 performs a negative regulation of prebound astrocyte-specific genes and efficiently hinders SOX9 from activating their expression (Klum et al., 2018). Astrocyte-specific genes become additionally targeted and activated by SOX9, while oligodendrocyte-specific genes are prebound by SOX9 only and later on they are targeted and activated by SOX10 during oligodendrocyte maturation (Figure 2) (Klum et al., 2018). Thus, the previous study demonstrated how sequentially expressed SOX proteins act on lineage-specific regulatory DNA elements to coordinate glial gene expression both in a temporal and in a sub-lineage-specific fashion (Klum et al., 2018).

Together these interesting data demonstrated that sequentially acting SOX TFs orchestrate neural lineage development including neuronal-, astrocyte- and oligodendrocyte-specific gene expression.

## The Roles of SOX Transcription Factors in Neuronal Differentiation

**FIGURE 3:**
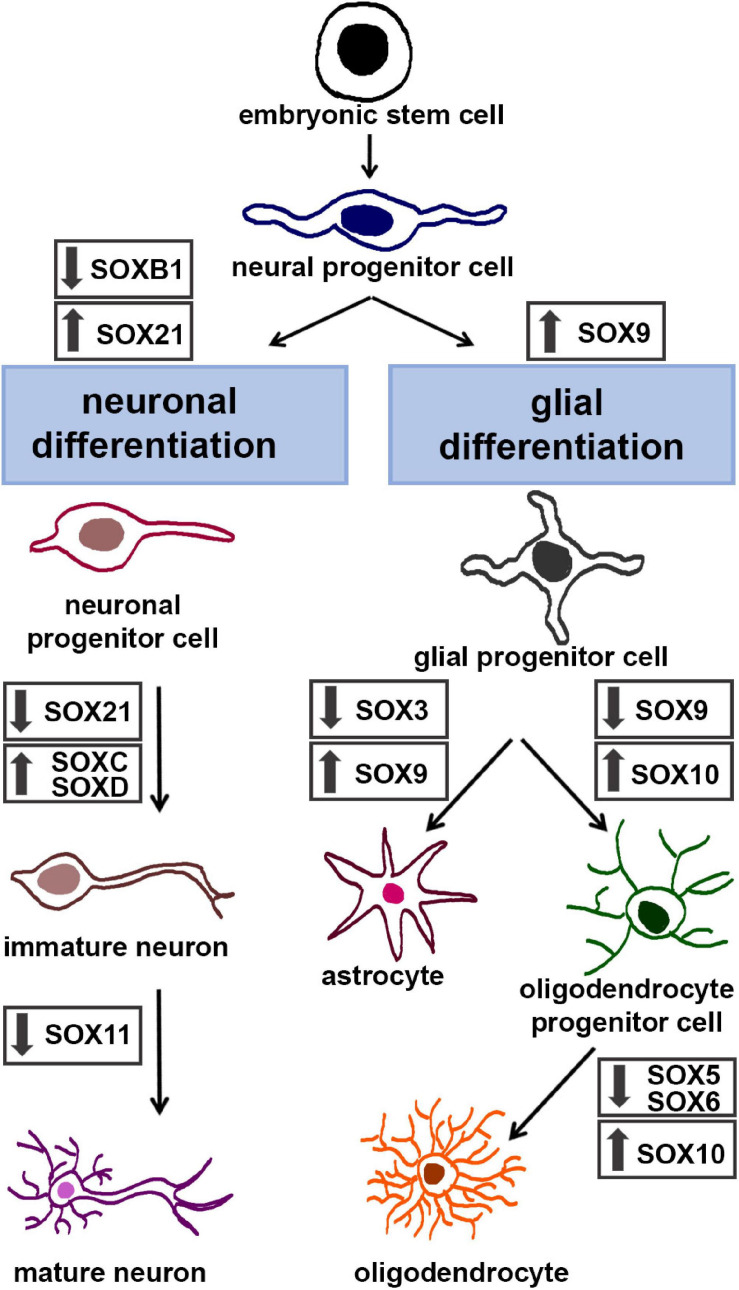
The roles of SOX TFs in neuronal and glial differentiation. Schematic illustration of stepwise neuronal differentiation process **(left)** and glial differentiation process **(right)**. SOX TFs expression levels during specific lineage-restricted progressions are presented by down-arrows (down-regulation) and up-arrows (up-regulation). The figure summarizes following data: for neuronal differentiation – (Connor et al., 1995; Bylund et al., 2003; Stolt et al., 2003; Sandberg et al., 2005; Wang et al., 2005; Bergsland et al., 2006; Batista-Brito et al., 2009; Martinez-Morales et al., 2010; Hoshiba et al., 2016; Naudet et al., 2018); for glial differentiation – (Stolt et al., 2003, 2004, 2006; Kellerer et al., 2006; Stolt and Wegner, 2010; Kang et al., 2012; Weider et al., 2013; Klum et al., 2018).

Neuronal differentiation is a complex process that relies on a timely and spatially controlled expression of transcriptional regulators (Rea et al., 2020). Numerous SOX TFs play widespread roles from initial phases of differentiation until generation of mature neurons (Avilion et al., 2003; Bylund et al., 2003; Graham et al., 2003; Bergsland et al., 2006; Hoser et al., 2008). During neuronal differentiation SOXB and SOXC members act sequentially (Bergsland et al., 2011). *SoxB1* genes are expressed in the neural precursor cells, while *Sox21*, *Sox4*, and *Sox11* are mostly expressed in neural cells committed to neuronal differentiation (Figure 3, left panel) (Uwanogho et al., 1995; Cheung et al., 2000).

SOXB1 proteins are necessary for formation of neuroectoderm, maintenance of the neural progenitor state and suppression of neuronal differentiation (Figure 3, left panel) (Bylund et al., 2003). It has been revealed that forced expression of *SoxB1* genes maintains neural cells in un undifferentiated state and inhibits neuronal differentiation, whereas their suppression induces upregulation of post-mitotic neuronal markers (Bylund et al., 2003). Down-regulation of *SoxB1* gene expression by Ngn2 (Neurogenin 2) is essential for neuronal differentiation (Bylund et al., 2003).

SOX1 is one of the earliest TFs expressed in cells committed to the neural fate. Its expression correlates with the formation of neural plate, while down-regulation of *Sox1* expression in the developing neural tube correlates with the exit of cells from mitosis (Pevny et al., 1998). Overexpression of *Sox1* in NPCs is sufficient to promote neuronal lineage commitment (Kan et al., 2004) while the loss of neurons in the ventral striatum was detected in the brains of *Sox1* null mutant mice (Malas et al., 2003).

*Sox2* expression is detected in the early neuroectoderm (Collignon et al., 1996) and SOX2 transcription factor is necessary to maintain neural progenitor populations throughout the developing central nervous system (CNS) (Hutton and Pevny, 2011). Constitutive *Sox2* expression kept NPCs in a precursor state and inhibited neuronal differentiation, while expression of a dominant-interfering form of *Sox2* led to exit from cell cycle, delamination of NPCs from the ventricular zone, loss of expression of progenitor markers and initiation of neuronal differentiation (Graham et al., 2003). We showed that constitutive *SOX2* overexpression altered expression of neuronal markers and reduced number of mature MAP2 (Microtubule Associated Protein 2) positive neurons upon RA induced neural differentiation of NT2/D1 cells (Drakulic et al., 2012; Klajn et al., 2014).

Expression of *Sox3* is detected throughout the developing CNS (Wood and Episkopou, 1999) and activity of this gene is necessary for formation of the hypothalamo–pituitary axis (Rizzoti et al., 2004). Ectopic *Sox3* expression in zebrafish led cells of the ectoderm to acquire a neural fate, while reduction of neural ectoderm was seen in *Sox3* knocked-down study (Dee et al., 2008).

*Sox21* expression is detected in NPCs (Ohba et al., 2004) while *Sox14* is expressed in limited population of neurons in the developing brain and spinal cord (Hargrave et al., 2000). The balance of *SoxB1* and *SoxB2* expression determines whether NPCs remain as progenitors or become committed to differentiation (Figure 3, left panel). SOX21 promotes neuronal differentiation by counteracting the activity of SOXB1 (Sandberg et al., 2005). Study conducted on *Xenopus laevis* shows that *Sox21*, like *Sox2*, functions in a dose-dependent manner and that its level of expression determines the decision between maintenance of neural progenitors and formation of neurons (Whittington et al., 2015). Namely, Whittington et al. (2015) proposed model which described how level of *Sox21* expression regulates progression of NPCs during neurogenesis. When *Sox21* expression is severely reduced, NPCs undergo cell death; with a minimal level of *Sox21* expression NPCs differentiate to become neurons while higher expression of *Sox21* inhibits neurogenesis, promotes *SoxB1* expression and progenitor maintenance (Whittington et al., 2015). On the other side, overexpression of *Sox21* in the chick neural tube led to reduction of cell proliferation, downregulation of *Sox3* expression and initiation of premature differentiation of NPCs (Sandberg et al., 2005). Furthermore, the maintenance of *Sox21* expression in NPCs disabled their terminal differentiation (Sandberg et al., 2005). Interestingly, recent study shows that both SOX21 and SOX14 have their own unique gene targets and therefore these TFs do not compete for the same target genes (Makrides et al., 2018). Thus, SOX21 is important for the maintenance, while SOX14 is necessary for terminal differentiation of the GABAergic neurons in the mouse brain (Makrides et al., 2018).

When NPCs start to differentiate into immature neurons, pro-neural proteins induce expression of SOXC TFs (Figure 3, left panel) (Bergsland et al., 2006). SOXC TFs are necessary to ensure survival of NPCs (Bhattaram et al., 2010) and for establishment of their neuronal properties (Bergsland et al., 2006). Overexpression of *Sox4* and *Sox11* led to premature induction of neuronal markers (Bergsland et al., 2006) while deficiency of both factors induced apoptosis in the developing nervous system (Bhattaram et al., 2010; Thein et al., 2010). Furthermore, reduced level of *Sox4* and *Sox11* resulted in reduced number of mature neurons and decreased neurite length (Chen et al., 2015). Also, results obtained by Hoshiba et al. (2016) indicate that high level of SOX11 expression, detected only in cortical neurons until birth, is necessary to suppress dendritic morphogenesis during radial migration.

As already pointed out, SOXB and SOXC members are sequentially bound to a common set of neural genes during the process of neuronal differentiation (Figure 2) (Bergsland et al., 2011). It has been found that 92% of the SOX3 binding sites will be targeted by SOX11 in newly formed neurons (Bergsland et al., 2011).

SOXD members are also involved in the process of neuronal differentiation (Figure 3, left panel). They are expressed in proliferating progenitors in the ventricular and subventricular zones and in post-mitotic neurons (Stolt et al., 2006; Azim et al., 2009; Lee et al., 2014; Quiroga et al., 2015). *Sox5* promotes exit of neural progenitors from cell cycle and downregulation of its expression is necessary for the progression of neuronal differentiation (Martinez-Morales et al., 2010). Furthermore, SOX5 post-mitotically controls the neuronal migration, molecular identity and subcortical axonal projections of subplate and deep-layer neurons (Kwan et al., 2008). This TF is involved in the control of neurite outgrowth (Naudet et al., 2018). SOX6 is important for positioning and maturation of cortical interneurons (Connor et al., 1995; Batista-Brito et al., 2009). *Sox13* is expressed in a sub-population of post-mitotic differentiating neuronal cells and results obtained by Wang et al. (2005) suggest that this gene may have a role in the specification and/or differentiation of a specific subset of neurons in the developing CNS.

*Sox9*, *SoxE* member, is expressed in neural stem cells (NSCs) and gain- and loss-of-function studies indicated that *Sox9* was required for multipotentiality and maintenance of NSCs during development (Scott et al., 2010). On the other side, *Sox9* overexpression led to reduction in the number of neuronal progenitors and neurons during the spinal cord development (Vogel et al., 2020).

The majority of existing knowledge regarding the functions of *Sox* genes in the process of neuronal differentiation is obtained by conducted experiments in mice and other animal models. However, comparative transcriptome analyses point to differences in gene expression between the human and the rodent brain (Zeng et al., 2012; Silbereis et al., 2016). Thus, it would be interesting to investigate the roles of *SOX* genes in human neuronal differentiation using iPSCs and 3D brain organoids.

## SOX Transcription Factors and Neurodevelopmental Disorders

Recent literature data has revealed that *SOX* gene variants are associated with neurodevelopmental disorders (NDDs), characterized by impairment of neuronal function during brain development. *SOX* gene variants associated with NDDs are listed in Supplementary Table 1.

Contribution of S*OX* genes to NDDs is still not clear. Both deletions and duplications of *SOX3* gene were detected in patients with intellectual disability (Stevanovic et al., 1993; Laumonnier et al., 2002; Helle et al., 2013; Stagi et al., 2014; Arya et al., 2019). Also, *SOX3* missense variant was detected in proband with mild intellectual disability (Jelsig et al., 2018). Furthermore, it was found that *SOX4* heterozygous missense variants cause neurodevelopmental disease (Zawerton et al., 2019). On the other side, *SOX5* haploinsufficiency and its loss of function variant have been found in probands with intellectual disability (Lamb et al., 2012; Schanze et al., 2013; Nesbitt et al., 2015). Also, NDDs have been detected in individuals with heterozygous *SOX6* variants (Tolchin et al., 2020). Significant down-regulation of *SOX9* expression has been revealed in neural progenitors derived from Fragile X Syndrome ESCs (Telias et al., 2015) while downregulation of *SOX10* expression is detected in brains of patients with schizophrenia (Iwamoto et al., 2005). Heterozygous missense variants within the HMG box of *SOX11* gene are associated with intellectual disability (Tsurusaki et al., 2014) while polymorphisms in distal 3′ untranslated region of this gene are associated with susceptibility for schizophrenia (Sun et al., 2020).

All these data open a new avenue of research focused on discovering the roles of SOX TFs and their gene targets in NDDs, making them promising biomarkers and potential targets for future diagnostic and therapeutic strategies.

## The Roles of SOX Transcription Factors in Adult Neurogenesis

In the mammalian brain, generation of neurons and astrocytes from NSCs throughout postnatal and adult life is mainly observed in the subventricular zone (SVZ) of lateral ventricle and in the subgranular zone (SGZ) of the dentate gyrus in the hippocampus (reviewed in Gage, 2000; Ming and Song, 2011; Lim and Alvarez-Buylla, 2016). NSCs that reside in the SGZ generate dentate granular cells which play roles in learning, memory and pattern separation (Kaplan and Bell, 1983, 1984; Ming and Song, 2011), whereas NSCs from the SVZ give rise to neuroblasts which migrate in the rostral migratory stream and differentiate to olfactory bulb neurons (Lois and Alvarez-Buylla, 1993).

Similar to neurogenesis in embryonic brain, the generation of new neurons from adult NSCs consists of the sequence of events including proliferation, differentiation and maturation, which are controlled by environment-derived signals and precise changes in the gene expression. However, despite many similarities between NSCs from embryonic and adult brain, emerging evidence suggests profound differences in these two cell populations, such as proliferation rates, neurogenic potential and gene expression profiles (reviewed in Gotz et al., 2016; Obernier and Alvarez-Buylla, 2019). In addition, there are important differences between NSCs from two major adult neurogenic niches, SVZ and SGZ in cellular and molecular properties which may arise as result of external signals received from different environments (reviewed in Obernier and Alvarez-Buylla, 2019).

**FIGURE 4:**
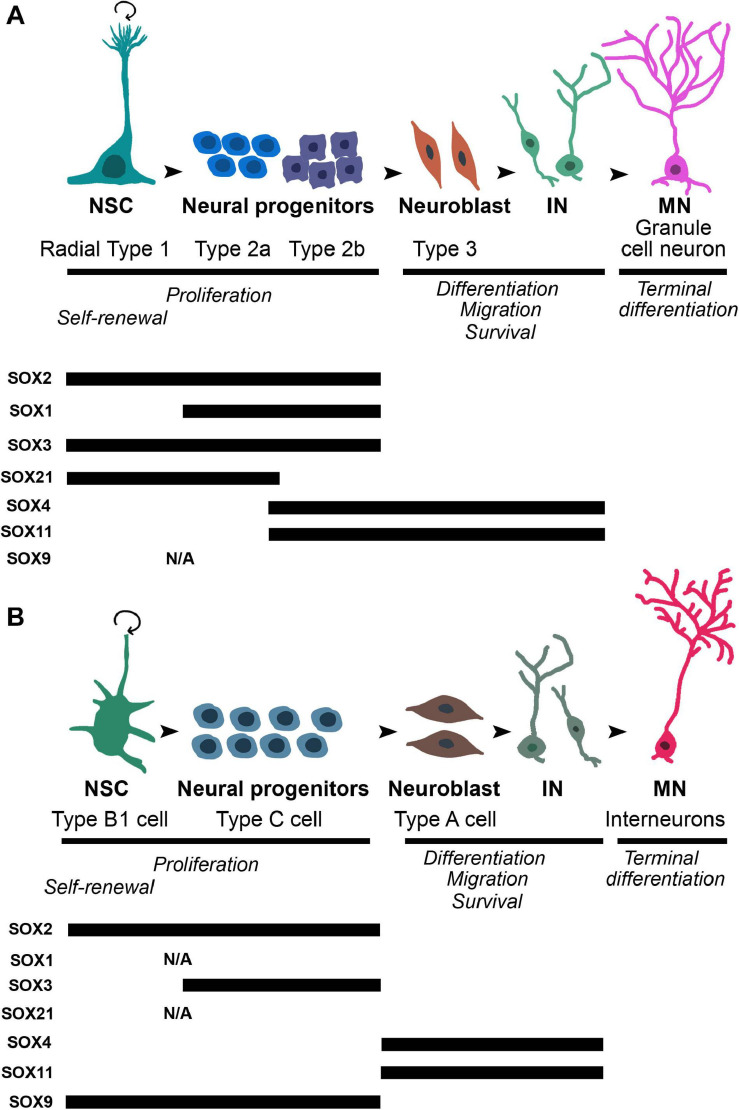
The dynamic expression profiles of selected SOX proteins during different stages of differentiation in neurogenic regions of adult brain: SGZ of dentate gyrus of the hippocampus and SVZ in the later wall of lateral ventricles. **(A)** Upper panel: the schematic illustration of the main stages of adult neurogenesis in SGZ. Radial type 1 cells, which correspond to neural stem cells, give rise to high proliferative Type 2 cells (first 2a, followed by 2b) which further generate Type 3 neuroblasts. Neuroblasts differentiate into granule neurons that migrate into granular cell layer of dentate gyrus. Lower panel: the schematic summary of the findings regarding the expression of SOX2 (Ferri et al., 2004; Steiner et al., 2006), SOX1 (Venere et al., 2012), SOX3 (Rogers et al., 2013), SOX21 (Matsuda et al., 2012), SOX4 (Mu et al., 2012), and SOX11 (Haslinger et al., 2009; Mu et al., 2012). **(B)** Upper panel: the schematic illustration of the main stages of adult neurogenesis in the SVZ. Neural stem cells, named as Type B1 cells upon activation generate high proliferative cell population of progenitors - Type C that give rise to Type A neuroblasts. Neuroblasts migrate through rostral migratory stream of the olfactory bulb where they differentiate into interneurons. Lower panel: the schematic summary of the findings regarding expression of SOX2 (Ferri et al., 2004), SOX3 (Rogers et al., 2013), SOX4 (Pennartz et al., 2004), SOX11 (Haslinger et al., 2009), and SOX9 (Cheng et al., 2009). NSC, neural stem cell; IN, immature neuron; MN, mature neuron; N/A, data not available.

The roles of SOX TFs in the regulation of adult neurogenesis, particularly in the hippocampus, are extensively investigated, with the main focus on SOXB, SOXC, and SOXD proteins (Figure 4) (reviewed in Wegner, 2011; Beckervordersandforth et al., 2015; Reiprich and Wegner, 2015). In the SGZ (Figure 4A) and SVZ (Figure 4B) of the mouse adult brain, SOX2 is mostly expressed in both, quiescent NSCs and highly proliferating multipotent neuronal progenitor cells (Ellis et al., 2004; Ferri et al., 2004; Steiner et al., 2006). Functional studies demonstrated that repression of *Sox2* gene expression impaired neurogenesis in the adult mouse brain (Ferri et al., 2004; Favaro et al., 2009; Amador-Arjona et al., 2015). SOX2 inhibits expression of pro-neurogenic TF NEUROD1 (Neuronal differentiation 1) via Wnt (Wingless-integration site)-signaling pathway, thus preventing neuronal differentiation and maintaining stem cells in a multipotent state (Kuwabara et al., 2009).

In contrast to SOX2, the expression of SOX1 has not been detected in NSCs, but it is revealed only in the highly proliferating neuronal progenitors of SGZ in the mouse adult hippocampus (Type 2a and Type 2b in the Figure 4A) (Venere et al., 2012). In the mouse adult brain, SOX3 protein has been detected in cells within neurogenic niches, however, with different pattern of expression. In the SGZ, its robust expression was identified in slow dividing NSCs as well as in highly proliferating Type 2a and Type 2b neuronal progenitor cells (Figure 4A) (Rogers et al., 2013). In contrast, the expression of SOX3 was localized only in small population of SOX2 expressing cells in SVZ (Figure 4B) (Rogers et al., 2013). Despite robust expression of SOX1 and SOX3 in adult brain, their functional roles in the regulation of adult neurogenesis are yet to be determined.

In the hippocampus of adult mouse, the expression of SOX21 has been detected in NSCs (Radial Type 1) and subset of neuronal progenitor cells (Type 2a) in SGZ (Figure 4A). Functional study indicated that *Sox21* regulates the progression of adult neurogenesis in the hippocampus by direct repression of TF Hes5 (Hes Family bHLH Transcription Factor 5) (Matsuda et al., 2012).

In the adult mammalian brain, the expression of SOX4 and SOX11 proteins is detected prominently in the both main neurogenic niches, SGZ (Figure 4A) and SVZ (Figure 4B) (Pennartz et al., 2004; Haslinger et al., 2009; Mu et al., 2012). Similar to the expression pattern in embryonic brain, onset of SOX4 and SOX11 expression in cells coincides with the down-regulation of SOX2 and up-regulation of DCX (Doublecortin) expression, and remains throughout the period when newborn neurons migrate to their final destination (Figure 4). Finally, SOX4 and SOX11 expression was detected in extremely low number of mature neurons (Haslinger et al., 2009; Mu et al., 2012). Overexpression of these two TFs promoted expression of neuronal-specific genes in NPCs, whereas their repression disturbed neurogenesis, but not gliogenesis (Mu et al., 2012).

Among SOXE TFs, SOX9 expression has been detected in the NSCs and different subset of neuronal progenitor cells in the SVZ in the adult mouse brain (Type B1 and Type C neuronal progenitors in the Figure 4B) (Cheng et al., 2009). Functional studies provided evidence that this TF is necessary for maintaining the multipotency of NSCs in SVZ (Scott et al., 2010). Furthermore, knockdown of *miR-124*, which targets *Sox9*, increase SOX9 expression and decrease neurogenesis (Cheng et al., 2009).

## SOX Transcription Factors and Impaired Adult Neurogenesis

Adult neurogenesis has been implicated as a major contributor of brain homeostasis, restoring neurological functions under physiological or pathological conditions (Kempermann et al., 2004; Braun and Jessberger, 2014). Differentiation and maturation of new neurons from NSCs in adult brain are dynamically regulated by numerous intrinsic and extrinsic factors, such as neurotrophic factors, transcriptional programs, inflammatory cytokine, cell cycle regulators, neurotransmitters and hormones (Braun and Jessberger, 2014; Shohayeb et al., 2018). On the other hand, the most studied negative regulators of NSC fate during adult neurogenesis include aging, stress, inflammation and alcohol abuse (Braun and Jessberger, 2014). In addition, wide spectrum of neurological conditions is a consequence of neuron loss after injury. Although increase in neurogenesis is detected in response to injury, the capacity for restoring of neurological function in damaged areas is limited (review in Dillen et al., 2020).

Neurodegenerative diseases are heterogeneous group of deleterious conditions with multifactorial etiologies caused by progressive damage of neurons and glial cells and, consequently, the loss of cognitive and physical functions. Recent findings provided multiple evidence for deregulated adult neurogenesis in several neurodegenerative diseases that display symptoms related to hippocampal and olfactory dysfunction, including Parkinson’s, Alzheimer’s, and Huntington diseases (reviewed in Deierborg et al., 2007; Winner et al., 2011; Winner and Winkler, 2015; Horgusluoglu et al., 2017).

Despite numerous data implicating the key role of *Sox*/*SOX* genes in regulation of embryonic and adult neurogenesis, their function under pathological conditions are largely unknown. Alzheimer’s disease, the most common adult onset-dementia, is characterized by deteriorating hippocampus, memory impairment, and other cognitive and olfactory deficits. Recently, Briley et al. (2016) have demonstrated reduction in SOX2 positive NSCs in the hippocampus of Alzheimer’s disease patients which correlated with the severity of the disease or the patient’s cognitive capacity.

In our previous work, we analyzed the expression of selected members of SOXB group (SOX1, SOX2, and SOX21) in the hippocampus of 2 months old 5xFAD mice, which represent a transgenic model of Alzheimer’s disease. Immunohistochemical analysis revealed a significant decrease in the number of cells expressing SOX1, SOX2, and SOX21 TFs within the SGZ of 5xFAD mice in comparison to their non-transgenic counterparts. Our comparative study also revealed, for the first time, significant difference in the number of SOX1 positive cells between genders in both, transgenic and non-transgenic animals (Zaletel et al., 2018). Considering previous findings that epigenetic-related mechanisms are involved in brain development (McCarthy and Nugent, 2015), we speculate that sex dependent level of SOX1 expression could be result of epigenetic regulation. However, further studies are needed to clarify these findings.

## The Roles of SOX Transcription Factors in Glial Differentiation

During embryonic development of the CNS, multipotent neural precursor cells undergo a characteristic temporal pattern of differentiation wherein neurons are generated ahead of the production of glial cells. This developmental transition consists of two distinct molecular processes: the termination of neurogenesis and the initiation of gliogenesis. This developmental interval, often named “gliogenic switch,” is fundamental to the entire developing CNS and is conserved throughout all the vertebrate species (Kessaris et al., 2001; Poche et al., 2008; Subramanian et al., 2011; Kang et al., 2012). The genesis of neural cells by chronological order in vertebrates provides a good biological sense. In fact, the generated neurons create the functional neuronal circuits and, when the scaffold is formed, then the numbers and positions of glia are fitted in the preformed platform (Miller and Gauthier, 2007). The integrated glial cells further provide mechanical, metabolic and trophic support to neurons. The sequential production of neurons and glia is best characterized in the ventral region of the mouse and chick embryonic spinal cord (Kessaris et al., 2001; Rowitch, 2004; Kang et al., 2012). SOX9 TF has been reported as a crucial molecular component in triggering the switch from neurogenic to gliogenic program in the developing mouse spinal cord (Stolt et al., 2003; Kang et al., 2012). In particular, the absence of *Sox9* in NSCs caused defects in the specification of oligodendrocytes and astrocytes, the two main types of glial cells in CNS, and induced a transient increase in the number of motoneurons (Stolt et al., 2003). However, findings in the developing cerebellum indicate that the primary functional role of *Sox9* in modulating the neuron-versus-glia switch is to suppress neurogenesis, rather than to actively trigger the initiation of gliogenesis (Vong et al., 2015). The discrepancy in results obtained by studying different developmental CNS regions suggests that the importance of SOX9 transcriptional factor in orchestrating NSCs fate decision toward gliogenesis may be tissue or organ dependent (Vong et al., 2015). During gliogenic switch SOX9 actively promotes glial lineage progression by controlling a set of genes that contribute to early gliogenesis (Kang et al., 2012). As previously mentioned, an important feature of SOX proteins is that they generally display their gene regulatory functions by forming complexes with partner TFs (Kamachi and Kondoh, 2013). Thus, the NFIA (Nuclear factor-1 A) directly regulated by SOX9, has been identified as the crucial transcriptional partner of SOX9 necessary for the onset of gliogenesis. Subsequently, SOX9 and NFIA form a complex and co-activate multiple genetics programs that regulate the activities of astroglial precursors (Kang et al., 2012). It is important to note that this data has been collected from the embryonic chick and mouse spinal cord, whether the same mechanism is active in the other regions of developing CNS is still unknown. Recent findings have indicated that the synergistic activation of astrocyte genes by SOX9 and NFIA is repressed by SOX3 binding in glial precursor cells of mouse spinal cord (Klum et al., 2018). Indeed, while both astrocyte- and oligodendrocyte-specific genes are prebound by SOX9 in glial progenitor cells, a specificity of astrocyte genes is that this prebinding occurs in combination with SOX3. *Sox9* continues to be expressed in maturing astrocytes but its expression decreases during oligodendrocyte-lineage progression (Figure 3, right panel). At the later stages of development, maturing oligodendrocytes become fully dependent on *Sox10*, as evident from the severe disruption of both terminal oligodendrocyte differentiation and myelination in the CNS of *Sox10*-deficient mice (Stolt et al., 2002; Weider et al., 2013).

Although *Sox8* is also expressed in oligodendrocyte precursors, it performs only supportive role during oligodendrocyte development (Stolt et al., 2004; Kellerer et al., 2006; Stolt and Wegner, 2010). Despite functional redundancy, SOX8 is only able to partially rescue the compromised oligodendrocyte differentiation in *Sox10*-deficient mice (Kellerer et al., 2006). However, differentiated oligodendrocytes rely to a much greater extent on SOX8 than oligodendroglial precursors, since recent findings have indicated that SOX8 and SOX10 are jointly required for maintaining the myelinated state (Turnescu et al., 2018).

SOX10 directly controls the expression of genes encoding the major myelin proteins (Stolt and Wegner, 2010). This TF is an essential general determinant of myelination in both CNS and the peripheral nervous system (Stolt and Wegner, 2010; Weider et al., 2013). Although both oligodendrocytes in CNS and Schwann cells in peripheral nervous system represent myelinating glia, they achieve myelination in distinct ways. However, while the interacting partners of SOX10 in Schwann cells are well described, less is known about its transcription partners in oligodendrocytes. Expression of *Sox5* and *Sox6* overlaps strongly with SOXE protein activity during oligodendrocytes specification and terminal differentiation (Figure 3, right panel). Nevertheless, while SOX9 and SOX10 promote oligodendrocyte lineage progression, SOX5 and SOX6 have the opposite effects as evident from both premature specification and precocious terminal differentiation of oligodendrocyte precursors in *Sox5/Sox6*-deficient mice (Stolt et al., 2006). Direct physical interaction between the SOXD and SOXE TFs during oligodendrocyte differentiation has not been reported. Actually, the SOXD proteins counteract the SOXE proteins by competition for the same binding sites and the recruitment of co-repressors to target gene promoters (Stolt and Wegner, 2010).

## SOX Transcription Factors in Tissue Homeostasis and Regeneration in the Context of Glial Cells

Interestingly, growing evidence indicates that *Sox* genes also play additional roles in adult tissue homeostasis and regeneration (Parrinello et al., 2010; Sarkar and Hochedlinger, 2013; Chen et al., 2019). However, in comparison to the data about the roles of *Sox* genes during developmental, this research field is less explored. Unlike neurons and oligodendrocytes, which become post-mitotic and take on a distinct morphology upon terminal differentiation, astrocytes are not permanently post-mitotic. In response to injury, these cells transform from quiescent into reactive state and dedifferentiate to a progenitor cell-like state (Buffo et al., 2008), which serves as a compensatory response that modulates tissue damage and recovery (Barreto et al., 2011). The re-expression of *Sox* genes in reactivated astrocytes of adult mouse brain has been reported (Bani-Yaghoub et al., 2006; Sun et al., 2017; Chen et al., 2019), but the better understanding of their roles in this process is still needed. Peripheral nerve regeneration is a good example for the role of *Sox2* gene in tissue repair. Upon injury, mature adult Schwann cells re-express *Sox2* and re-acquire progenitor cell-like characteristics (Parrinello et al., 2010). *Sox2* re-expression seems to play a direct role in Schwann cell clustering, a key event during nerve regeneration that enables Schwann cells to form multicellular cords to guide axon re-growth across the site of injury (Parrinello et al., 2010).

The link between neural functions of *SOXE* genes and human nervous system pathologies has been reported throughout different studies. Thus, the increased number of SOX9- and SOX10-positive early glial progenitors in brains of multiple sclerosis patients has been reported (Nait-Oumesmar et al., 2007). Additionally, *SOX8* has been identified as genetic risk loci for Multiple Sclerosis in humans (International Multiple Sclerosis Genetics Consortium et al., 2011, 2013). Also, dysfunction of oligodendrocytes in patients with schizophrenia was correlated with increased DNA methylation of *SOX10* gene (Iwamoto et al., 2005). Taking together, these data imply that *SOX* genes could be considered as potential therapeutic targets. Modulation of *SOX* genes expression may change the functional properties of glial cells for more efficient remyelination of neurons or the repopulation of damaged areas upon CNS trauma. Full understanding of *SOX* genes function in nervous system development as well as homeostasis maintenance and regeneration holds the promise for development of novel therapeutic strategies.

## Cross-Talk of SOX Transcription Factors With Wnt/β-Catenin and RA Signaling Pathways

During last decade numerous results established SOX TFs as key players in various signaling pathways. Their interplay with Wnt/β-catenin and RA signaling pathways is of particular interest since SOXB1 neural-specific interpretation of these signaling cascades are involved in the maintenance of stemness and neural differentiation (Kormish et al., 2010; Oosterveen et al., 2013; Hagey and Muhr, 2014).

## SOX Interplay With Wnt/β-Catenin Signaling

β-catenin is central signaling molecule in canonical Wnt pathway (Clevers, 2006). In the absence of the Wnt ligand, cytosolic β-catenin level is low due to activation of destruction complex (Orford et al., 1997; Salic et al., 2000). Wnt stimulation results in the inhibition of cytosolic β-catenin degradation and its shuttle to nucleus (Gordon and Nusse, 2006). In the nucleus β-catenin interacts with the TCF/LEF (T cell factor/Lymphoid enhancer factor) family of DNA-binding TFs on Wnt Response Elements and enhances expression of Wnt target genes (Gordon and Nusse, 2006; Fiedler et al., 2015).

Both canonical Wnt signaling and SOXB1 proteins promote self-renewal of NPCs (Kamachi et al., 1998; Pevny and Placzek, 2005; Wang et al., 2006; Kiefer, 2007). In addition, SOXB1/β-catenin interplay fine tunes the complex mechanism involved in pluripotency/differentiation switch. Activation of canonical Wnt signaling enhances self-renewal of mouse and human ESCs and embryonal carcinoma cells (Hanna et al., 2010; Hassani et al., 2012; Mojsin et al., 2015).

**FIGURE 5:**
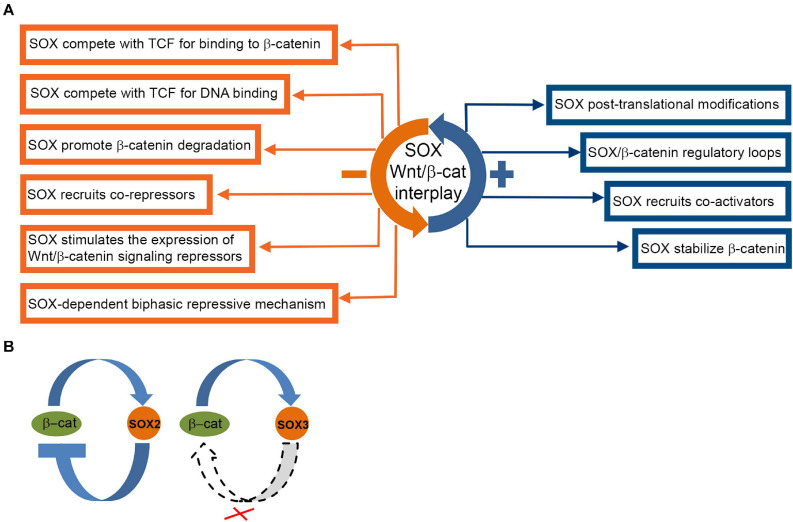
Complex interactions of SOX proteins and Wnt/β-catenin signaling pathway. **(A)** Overview of SOX interactions with Wnt/β-catenin pathway (Bernard and Harley, 2010; Kormish et al., 2010; Hagey and Muhr, 2014). SOX protein repression and activation mechanisms are indicated by orange and blue boxes, respectively. **(B)** SOX2 and SOX3/β-catenin crosstalk in NT2/D1 cells. Proposed model of mutual regulation of SOX2 and SOX3 and β-catenin in NT2/D1 cells (Mojsin et al., 2015). Crossed dashed lines indicate absence of regulatory link.

In neural progenitors Wnt/β-catenin signaling activates expression of the pro-neural gene *NeuroD1* by counteracting SOX2-mediated repression on DNA element containing overlapping SOX2 and TCF/LEF-binding sites (SOX/LEF) in its promoter (Van Raay et al., 2005; Kuwabara et al., 2009). We showed that lithium induced activation of Wnt/β-catenin signaling increased expression of all SOXB1 proteins in NT2/D1 cells. We also demonstrated that increase in SOX2 and SOX3 protein expression is β-catenin dependent, while overexpression of SOX1 is governed by β-catenin-independent manner (Figure 5B) (Mojsin et al., 2015).

SOX1, SOX2, and SOX3 also affect Wnt/β-catenin signaling (Zorn et al., 1999; Zhang et al., 2003; Kan et al., 2004). SOX1 binds to β-catenin via their C-terminal regions (Akiyama et al., 2004) and inhibits β-catenin/TCF transcription activity in mouse and human NPCs in the onset of neural differentiation (Kan et al., 2004). *SOX2* overexpression reduced β-catenin protein level and down-regulated Wnt/β-catenin signaling in NT2/D1 cells, suggesting negative feedback loop between β-catenin and SOX2 (Figure 5B) (Mojsin et al., 2015). However, overexpression of *SOX1* and *SOX3* genes has no effect on endogenous β-catenin level in NT2/D1 cells (Figure 5B) (Mojsin et al., 2015).

SOX3 down-regulates Wnt signaling by interactions with β-catenin or by direct binding to the regulatory regions of Wnt target genes (Zorn et al., 1999; Zhang et al., 2003). SOX21 restricts Wnt activity by interacting with β-catenin and subsequent interfering with the binding of TCF4/β-catenin complex to the *WNT8B* (Wingless-type MMTV integration site family, member 8B) enhancer (Fang et al., 2019).

The SOX proteins are considered as nuclear regulators of β-catenin/TCF activity responsible for fine tuning of transcriptional responses to Wnt signaling (Kormish et al., 2010). They regulate β-catenin/TCF activity by recruitment of various mechanisms in cell context-dependent manner (review in Kormish et al., 2010). SOX proteins can physically interact with both β-catenin (Zorn et al., 1999; Akiyama et al., 2004; Iguchi et al., 2007; Sinner et al., 2007; Bernard and Harley, 2010) and TCF/LEF (Sinner et al., 2007). SOX and TCF bind to similar DNA sequences in the DNA minor groove and induce DNA bending which enables assemble of SOX/TCF complexes regardless of distance of their binding sites (Bernard and Harley, 2010; Hou et al., 2017). Post-translational modifications of SOX, β-catenin and TCF also affect their interactions (Taylor and Labonne, 2005; Arce et al., 2006; Hattori et al., 2006). SOX proteins bind to the promoters of Wnt target genes and recruit transcriptional co-activators or co-repressors thus controlling β-catenin dependent transcriptional activity (Tsuda et al., 2003; Zhang et al., 2003; Furumatsu et al., 2005; Iguchi et al., 2007; Pan et al., 2009). SOX proteins activate the expression of Wnt signaling pathway repressors (Bastide et al., 2007). In addition, SOX proteins control endogenous β-catenin protein level by promoting either, proteosome-mediated β-catenin degradation (Preiss et al., 2001; Sinner et al., 2007; Guo et al., 2008) or its stabilization (Figure 5A) (Sinner et al., 2007).

Wnt and SOX interplay and mutual control led to a hypothesis pointing out that interactions between lineage-specific SOX TFs and β-catenin/TCF govern specificity of Wnt/β-catenin dependent transcription (Mukherjee et al., 2020). This idea is supported by the study conducted by Hagey and Muhr (2014). They studied the transition between stem cells and rapidly dividing progenitors in mouse cortex and proposed model of SOXB1-dependent bi-phasic repressive mechanism (Hagey and Muhr, 2014). High level of SOXB1 in stem cells represses pro-proliferative genes, primarily *Ccnd1* (Cyclin D1), by binding to low-affinity SOX binding sites in *Ccnd1* promoter, by interactions with TCF/LEF proteins and by recruitment of GRO/TLE (Groucho/Transducin-like Enhancers) co-repressors. Upon differentiation, pro-neural proteins reduce SOXB1 level, thus only the high affinity SOX binding sites stay occupied, while loss of binding to low-affinity sites de-represses *Ccnd1* and promotes proliferation of progenitor cells (Hagey and Muhr, 2014). Proposed model provides the explanation how presence of low- and high-affinity SOX binding sites enables graded SOXB1 target gene regulation and how differences in the expression levels of SOXB1 proteins can be interpreted by determining the response of target genes.

It has been shown that complex network between Wnt/β-catenin pathway, SOX2 and proneural genes regulates the progression from progenitors to neurons and glia cells (Agathocleous et al., 2009). Lack of this coordination leads to aberrant neuronal proliferation and differentiation and contributes to the pathology of psychiatric disorders (Agathocleous et al., 2009; Santos et al., 2021). In the search for specific targets of lithium resistance in the patients with bipolar disorder, Santos et al. (2021) conducted comparative transcriptome analysis of the hippocampal dentate gyrus-like neurons derived from iPSCs of lithium-responsive and lithium-non-responsive patients. First, they have demonstrated that neurons generated from both cohorts exhibited neuronal hyperexcitability compared to control neurons that could be reversed by lithium treatment of the lithium-responsive neurons only (Santos et al., 2021). The study showed that neurons from lithium-non-responsive patients acquire distinct phenotypic characteristics, electrophysiological properties and the response to lithium during differentiation due to the severely affected function of canonic Wnt/β-catenin signaling with a significant decrease in expression of LEF1 (Lymphoid enhancer-binding factor 1) (Santos et al., 2021). Interestingly, SOX2 was also up-regulated in lithium-non-responsive neurons compared to control neurons (Santos et al., 2021).

## SOX Interplay With RA Signaling

Retinoic acid exerts its pleiotropic effects through binding to retinoic acid receptors (RARs), members of the nuclear receptor superfamily (reviewed by Rochette-Egly and Germain, 2009). RARs act in heterodimeric combinations with retinoid X receptors (RXRs). It was suggested that RXRs act as scaffolding proteins and facilitate DNA binding of the RAR-RXR complex (Chawla et al., 2001). In the nucleus, RAR/RXR dimers can interact with *cis*-acting RA response elements (RAREs) (Laudet and Gronemeyer, 2002), atypical RARE (Panariello et al., 1996; Brondani et al., 2002) and composite response units within the promoters of different RA-target genes (Redfern, 2004; Wang and Yen, 2004). Ligand binding induces conformational changes that lead to release of co-repressors, binding of co-activators and subsequent initiation of transcription (Balmer and Blomhoff, 2002).

**FIGURE 6:**
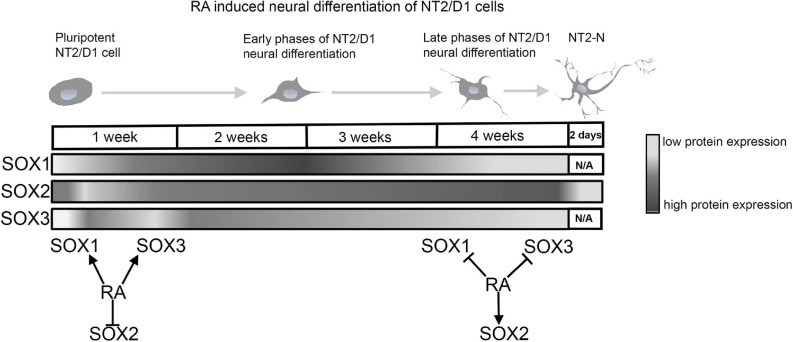
RA modulates expression of SOXB1 proteins during neural differentiation of NT2/D1 cells. Schematic representation of SOXB1 proteins expression profiles during 4 weeks of RA induced neural differentiation of NT2/D1 cells. RA has opposite effects on SOXB1 proteins expression during early and late phases of neural differentiation of NT2/D1 cells (Stevanovic, 2003; Klajn et al., 2014; Popovic et al., 2014; Topalovic et al., 2017). N/A, not available data.

Our group conducted comprehensive analyses of the SOXB1 protein expression during RA induced neural differentiation of NT2/D1 cells (Stevanovic, 2003; Klajn et al., 2014; Popovic et al., 2014; Topalovic et al., 2017). Obtained results showed dynamic changes in the expression profiles of SOX1, SOX2 and SOX3 proteins during 4-weeks course of RA induction (Figure 6).

Retinoic acid induced transient up-regulation of SOX1 at the day 4 of RA induction and oscillating expression followed by decrease at 3 and 4 weeks of treatment (Figure 6) (Popovic et al., 2014; Topalovic et al., 2017). After initial downregulation in the first 48h of induction, SOX2 was up-regulated in all time points of induction (Figure 6) (Stevanovic, 2003; Popovic et al., 2014; Topalovic et al., 2017). However, in mature neurons (NT2-N) expression of SOX2 is abolished (Klajn et al., 2014). Similar results were obtained in the studies of the effects of RA on *Sox2* expression in mouse P19 and F9 embryonal carcinoma cell lines (Wiebe et al., 2000; Tremblay et al., 2012; Popovic et al., 2014).

SOX3 expression was transiently up-regulated during 48h of RA treatment and then gradually decreased up to 4 weeks of RA treatment (Figure 6) (Stevanovic, 2003; Popovic et al., 2014; Topalovic et al., 2017). Comprehensive examination of the promoter of human *SOX3* gene revealed the presence of two RA response elements, DR-3-like RXR RE (Nikcevic et al., 2008) and atypical RA/RXR RE (Mojsin et al., 2006). In addition, we have identified numerous TFs involved in the modulation of RA induced activation of human *SOX3* promoter (Krstic et al., 2007; Nikcevic et al., 2008; Mojsin and Stevanovic, 2009).

Beside RA involvement in transcriptional regulation of SOXB proteins, SOXB1 neural-specific interpretation of signaling morphogens add an additional level of complexity to the RA/SOXB1 interplay in developing CNS (Oosterveen et al., 2013). Genome-wide characterization of *cis*-regulatory modules (CRMs) in neural-specific target genes (Oosterveen et al., 2013) showed that interpretation of pleiotropic signals is the result of integration of SOXB1 and signaling morphogens on CRMs (Oosterveen et al., 2013). CRMs of RA target genes contains RARE and SOX binding sites both required for synergistic activation of CRMs (Oosterveen et al., 2013). One of genes enriched for functions in neural development whose CRM was analyzed in the study is *Dbx1* (Developing brain homeobox 1) (Oosterveen et al., 2013). In another study conducted by Rogers et al. (2014) *Dbx1* was identified as a direct and exclusive SOX3 target gene in NPCs both *in vitro* and *in vivo*. The fact that RA regulates SOX3 expression through multiple RARE (Brunelli et al., 2003; Mojsin et al., 2006; Nikcevic et al., 2008) confirms that SOXB1/RA signaling interplay is complex and fine-tuned at multiple levels.

## SOX Transcription Factors and microRNAs in Control of Neuronal and Glial Differentiation

**FIGURE 7:**
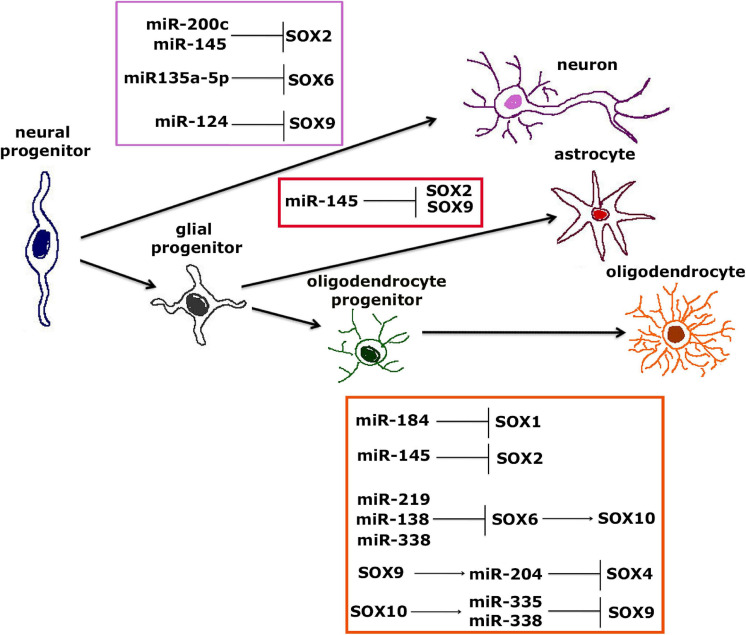
Interaction between SOX TFs and miRNAs during neural differentiation from neural progenitors to neurons, astrocytes or oligodendrocytes. Interaction between SOX TFs and miRNAs involved in differentiation of neurons from NPCs is shown within purple frame (Cheng et al., 2009; Peng et al., 2012; Cimadamore et al., 2013; Kamachi and Kondoh, 2013; Morgado et al., 2016; Li et al., 2019). Interaction between SOX TFs and miRNAs involved in differentiation of astrocytes from NPCs is shown within red frame (Krichevsky et al., 2006; Zhou et al., 2018), while interaction between SOX TFs and miRNAs involved in differentiation of mature oligodendrocytes from oligodendrocytes progenitors is shown within orange frame (Dugas et al., 2010; Zhao et al., 2010; Gokey et al., 2012; Hoffmann et al., 2014; Reiprich et al., 2017; Nazari et al., 2018; Afrang et al., 2019; Wittstatt et al., 2020).

Many evolutionary conserved microRNAs (miRNAs) present key factors in fine regulation of self-renewal and proliferation of NSCs and NPCs (Meza-Sosa et al., 2014). By interaction with complementary sequence motifs in 3′ untranslated region of target genes, miRNAs regulate the gene expression during different stages of neurogenesis, thus affecting the development of nervous system (Meza-Sosa et al., 2014). Also, acting in synergy with TFs, miRNAs form regulatory networks that can influence cell fate decision (Stappert et al., 2015). Therefore, it is not surprising that miRNAs are often called “master regulators” or “fine-tuners” of gene expression orchestrating important processes during neural development (Rajman and Schratt, 2017). SOX TFs and miRNAs represent one of the most important regulatory networks that control whether NSCs will self-renew or differentiate into neurons, astrocytes or oligodendrocytes (Figure 7) (Reiprich and Wegner, 2015). Particularly, SOX1, SOX2, SOX4, SOX5, SOX6, SOX9, and SOX10 are shown to interact with different miRNAs and orchestrate differentiation into neurons and oligodendrocytes (Figure 7).

It is suggested that miR-200 family members target *Sox2*, thus regulating the transition from NSCs to postmitotic and differentiated cells (Peng et al., 2012). It turns out to be one of the most important regulatory networks during neural differentiation, whereas *Sox2* is regulated by miR-200c, forming a negative-feedback loop that further decreases expression of *Sox2* during neural differentiation (Kamachi and Kondoh, 2013). Another axis shown to be important for neuronal differentiation is miR-135a-5p/*Sox6*/CD44, where miR-135a-5p acts through *So*x6, affecting not only differentiation of neurons, but also development of dendrites (Li et al., 2019). Moreover, it was shown that overexpression of *Sox6* could reverse miR-135a-5p-mediated neuronal differentiation and dendrite development of P19 cells (Li et al., 2019). *Sox9* is target of miR-124 during adult neurogenesis in the mouse SVZ where inhibition of *Sox*9 expression leads to differentiation into neurons (Cheng et al., 2009). Further, miR-145 is important for differentiation of neurons through regulation of *Sox2*–Lin28/let-7 signaling pathway that represents important mechanism for proliferation of NPCs (Cimadamore et al., 2013; Morgado et al., 2016). Additionally, miR-145 directly regulates *Sox2* and suppresses its expression in NPCs leading to induction of neurogenesis (Morgado et al., 2016).

Surprisingly, to the best of our knowledge, there is only one study suggesting that interaction between miRNA and SOX TFs plays role in differentiation of astrocytes. miR-124, with pivotal role in differentiation of neurons and astrocytes, can induce differentiation of NSCs to astrocytes through regulation of *Sox2* and *Sox9* expression in NSCs of amyotrophic lateral sclerosis transgenic mice (Krichevsky et al., 2006; Zhou et al., 2018).

Interestingly, it was shown that miR-184, which is one of the key miRNAs throughout all stages of oligodendrocytes differentiation, directly targets *Sox1*, leading to differentiation of NPCs to oligodendrocytes (Hoffmann et al., 2014; Afrang et al., 2019). During differentiation of NPCs the loss of *Sox2* also results in terminal differentiation of oligodendrocytes through negative regulation by miR-145 (Hoffmann et al., 2014). miR-219, miR-138 and miR-338 directly inhibit the expression of *Sox6* gene which results in reduced proliferation of oligodendrocyte progenitors and induction of oligodendrocyte differentiation and myelination (Dugas et al., 2010; Zhao et al., 2010). Further, SOX6 TF is directly regulated by miR-219 and additionally this TF represses the expression of *Sox10* in oligodendrocyte progenitor cells which results in differentiation of mature oligodendrocytes (Nazari et al., 2018). miR-204 overexpression also leads to differentiation of oligodendrocyte progenitors to mature oligodendrocytes through control of *Sox4* gene, while the expression of this miRNA is regulated by SOX9 (Wittstatt et al., 2020). SOX10 also regulates miR-338 that is important for differentiation of oligodendrocytes through inhibition of *Hes5* and *Hes6* genes and it is suggest that *Sox10* exerts its role in maturation of oligodendrocytes specifically through regulation of miR-338 (Gokey et al., 2012). Another study shows that SOX10 TF directly targets miR-338 and miR-335, and then these two miRNAs further repress *Sox9* in oligodendroglial cells (Reiprich et al., 2017). This fine tuning of *Sox9* and *Sox10* expression levels leads to terminal differentiation of oligodendrocytes.

It is evident that SOX TFs and miRNAs establish the functional interactions important for cell fate decision of NPCs during neural development. This can be achieved either through post-transcriptional regulation of *Sox* genes by miRNAs, through SOX-dependent control of miRNAs expression or by combination of both. Even though SOX3, SOX14, and SOX21 play important roles in neural differentiation, there is a lack of data regarding their regulation by miRNAs. Although SOX TFs are involved in regulation of gliogenesis, more studies are required to clarify posttranscriptional regulation of *Sox*/*SOX* gene expression by miRNAs during this process. Additionally, we previously reported that specific *SOX* genes and miRNAs can be potentially used as biomarkers for monitoring radiation response during early phase of neural differentiation (Stanisavljevic et al., 2019). This result suggests that SOX and miRNAs have additional important roles during neural differentiation. Overall, future studies are needed in order to gain better insight into the complex interactions between miRNAs and SOX TFs during neural development.

## Concluding Remarks and Future Directions

SOX proteins belong to the family of TFs that exerts multiple important roles during nervous system development, starting from preimplantation embryo to the adulthood. Many *Sox* genes are expressed in the developing and adult nervous system in the overlapping manners, covering various cell types, beginning with the neural stem cell (NSC) stage until terminal maturation of neurons and macroglia (reviewed in Lefebvre et al., 2007). During embryonic development SOX family members are involved, both in maintaining multipotency of neural progenitors, as well as in the promotion of neuronal differentiation (Table 2). They are also implicated in the control of glial differentiation by promoting differentiation of astrocytes and oligodendrocytes. SOX proteins may have dual roles in the regulation of target gene expression, acting as either activators or repressors, depending on cellular and genomic context (Liu et al., 2014). For instance, SOX3 is acting as activator of genes in neural progenitors, while suppressing neuronal differentiation by repression of neuronal and glial specific genes in the same cells (Figure 2). It is interesting to point out that SOXB and SOXC family members are sequentially bound to the common set of neural genes during the process of neuronal differentiation, highlighting the context-dependent nature of their actions. It has been reported that majority of the SOX3 binding sites will be targeted by SOX11 in newly formed neurons (Bergsland et al., 2011). The presence of low- and high-affinity SOX binding sites enables graded control of target genes, while the levels of SOX proteins is interpreted by the corresponding response of the target gene expression (Hagey and Muhr, 2014). Thus, sequentially acting SOX TFs orchestrate neuronal-, astrocyte- and oligodendrocyte-specific gene expression defining neuronal and glial phenotypes.

**TABLE 2:** Main functions of SOX TFs in neural differentiation during embryonic development.

Main functions in neural differentiation during embryonic development	SOX TFs	References
Maintainance of neural progenitor cells	SOX1, SOX2, SOX3, SOX9	Bylund et al., 2003; Graham et al., 2003; Scott et al., 2010; Hutton and Pevny, 2011
Promotion of neuronal differentiation	SOX14, SOX21, SOX4, SOX11, SOX5, SOX6, SOX13	Connor et al., 1995; Hargrave et al., 2000; Sandberg et al., 2005; Wang et al., 2005; Bergsland et al., 2006; Kwan et al., 2008; Martinez-Morales et al., 2010; Chen et al., 2015; Makrides et al., 2018
Promotion of astrocytes differentiation	SOX9	Stolt et al., 2003; Kang et al., 2012
Inhibition of astrocytes differentiation	SOX3	Klum et al., 2018
Promotion of oligodendrocytes differentiation	SOX8, SOX9, SOX10	Stolt et al., 2004; Stolt and Wegner, 2010; Weider et al., 2013
Inhibition of oligodendrocytes differentiation	SOX5, SOX6	Stolt et al., 2006

The capability of SOX TFs to orchestrate the process of neural differentiation strongly relies on epigenetic regulation. We already showed that *SOXB1* genes were controlled by different epigenetic mechanisms during neural differentiation (Topalovic et al., 2017). Additional study of the differences in histone signatures will provide further insight into the epigenetic regulation of pluripotency for proper differentiation of neurons or glial cells.

The roles of SOX proteins are not limited to development since these factors influence survival, regeneration, cell death and control of homeostasis in adult tissues (Pevny and Placzek, 2005). Adult neurogenesis has been recognized as a major contributor of brain homeostasis, restoring neurological functions under physiological or pathological conditions. We provide the overview of the current data implicating at least seven SOX proteins (members of SOXB, SOXC, and SOXE groups) in control of adult neurogenesis (Figure 4).

The majority of current knowledge regarding the roles of *Sox*/*SOX* genes in neural development is based on research mainly conducted in mice and other animal models, and to the lesser extent, on the *in vitro* cell based models of human neural differentiation. Research based on animal models provides important information about the roles of *Sox* genes in neural development. However, significant differences in brain development between species have been revealed and evident divergence among species is discovered regarding gene expression at the earliest stages of brain development (Johnson et al., 2009; Rakic, 2009; Clowry et al., 2010).

The emerging data associates *SOX* gene variants with NDDs characterized by impairment of neuronal function during brain development (Supplementary Table 1). Interestingly, down-regulation of *SOX9* expression has been detected in neural progenitors derived from Fragile X Syndrome human ESCs (Telias et al., 2015). However, contribution of SOX proteins to NDDs is still not fully explored and further research is needed to clarify their roles in the underlying pathologies.

The rising field of research is devoted to study the roles of SOX TFs in neurodegenerative diseases. Neurodegenerative diseases are characterized by progressive damage of neurons and glial cells and, consequently, loss of cognitive and physical functions. Recent years provided multiple evidences of impaired adult neurogenesis in several neurodegenerative diseases (reviewed in Horgusluoglu et al., 2017). Reduction in SOX2 positive NSCs detected in the hippocampus of Alzheimer’s disease patients is correlated with the severity of the disease or the patient’s cognitive capacity (Briley et al., 2016).

Most of the knowledge regarding human neurodegenerative diseases has been acquired from post-mortem patient samples since human brain tissue is inaccessible and highly difficult to obtain. Although many animal models mimicking diseases have been available for the research, they have provided only limited success in identification of the molecular mechanisms underlying human brain diseases. In the recent years, generation of patient-specific iPSCs provides remarkable opportunity to recapitulate both normal and pathologic human tissue formation *in vitro*, enabling genuine disease investigation (reviewed in Park et al., 2008). Furthermore, iPSCs have potential to replace affected neurons in neurodegenerative disorders (Comella-Bolla et al., 2020). Various human brain diseases across the spectrum of neurodevelopmental, neurodegenerative and neuropsychiatric are being studying by iPSC –based disease modeling (reviewed in McKinney, 2017). Studying the differentiation of patient-specific iPSCs into neurons or glial cells provides valuable insight into the molecular mechanisms underlying brain diseases in patient-specific genetic background. In the last decades remarkable efforts have been made in developing protocols for fast and efficient differentiation of iPSCs in specific neuronal sub-types. Recently, Comella-Bolla et al. (2020) described a fast, robust and reproducible protocol for differentiation of human iPSCs into functionally maturing forebrain neurons *in vitro*, which will facilitate studies of neurodevelopmental and neurodegenerative disorders. Obtained neurons have ability of *in vivo* integration which makes the protocol compatible with cell therapy-based strategies (Comella-Bolla et al., 2020). Apart from enabling research of disease phenotype *in vitro*, iPSCs are providing the tool for gene defect/s repair *ex vivo*. Moreover, iPSCs from healthy donors can be modified by introducing disease–specific mutation by genome editing allowing the study of the effect of specific gene defect in “healthy” background.

In an aging society, regenerative therapies based on iPSCs could provide significant potential therapeutic benefits, in particular for the patients suffering from neurodegenerative diseases including Parkinson’s and Alzheimer’s diseases. Study of the impacts of donor age on iPSC- derived cell functionality indicate that aging may reduce reprogramming efficiency having no significant effects on iPSCs maintenance or differentiation capacity (Strassler et al., 2018). These data suggest that donor age does not limit applications of iPSCs based methodology for modeling genetic diseases and for development of therapies for age-related diseases, especially in combination with recently developed gene-editing tools such as CRISPR/Cas9 technology (Strassler et al., 2018). The same authors indicate that burden of age-associated somatic mutations that iPSCs inherit from donor cells cannot be reduced, increasing the risk of abnormalities in iPSCs. A low number of healthy and elderly donors serving as a source of control cells present a great challenge in research and applications in the field of iPSCs (Strassler et al., 2018). Recently a collection of iPSCs derived from old male and female healthy subjects has been reported (Rodriguez-Traver et al., 2020) that can be used as controls for other disease lines derived from geriatric patients and for studying the roles of SOX TF in neurodegenerative disorders and aging.

Traditionally, brain diseases have been generally assigned to malfunction or loss of neurons. However, in the last decade, it has been shown that astrocytes play essential roles in the regulation of various brain functions. Astrocytes process and control synaptic information, modulate synaptic formation and elimination at all stages of development and in adulthood (Volterra and Meldolesi, 2005). Patient-specific iPSC-based models as human platforms for research accelerated the study of molecular mechanisms underlying neurogenesis, synapse formation, maintenance and plasticity (Oksanen et al., 2019). However, whether astrocytes contribute to the pathology of underlying brain disorders and potential contribution of SOX TFs to the pathologies is yet to be discovered. Accordingly, astrocytes became a promising target for drug discovery and the development of novel therapies.

Furthermore, wide spectrum of neurological and neurodegenerative conditions are consequence of neuron loss after ischemic injury. Although increase in neurogenesis is detected in response to injury, the capacity for restoring neurological function in damaged areas is limited (review in Dillen et al., 2020). Accordingly, iPSCs can be used for developing effective therapies aimed to increase neurogenesis by modulating *SOX* gene expression toward enhancement of regenerative potential for repair of damaged or aged neural cells.

Most recently advances in biotechnology, including stem cell propagation and novel biomaterials enable development of 3D models for studying human brain development. Brain organoids represent 3D-aggregates generated from human pluripotent cells (ESCs and iPSCs) resembling the embryonic human brain regarding the cell types, cells’ architectures and maturation (Eiraku et al., 2008; Pasca et al., 2015). The 3D models, ranging from region-specific organoids to more complex whole-brain organoids, are mimicking cell interactions and interconnectivity between multiple brain regions (Lancaster and Knoblich, 2014; Xiang et al., 2020) providing novel tools for studying more complex phenotypes involving different neuronal networks, tissue architecture, and organ morphogenesis (Baldassari et al., 2020). Advances in 3D modeling including extracellular matrix composition, optimized media transitions and agitation of the tissues led to the formation of cerebral organoids with various brain region identities. These advances revealed the remarkable fidelity with which organogenesis can occur *in vitro* leading to accurate modeling of events occurring during the first half of gestation in humans (reviewed in Chiaradia and Lancaster, 2020). While cerebral organoids are capable to spontaneously acquired forebrain, midbrain and hindbrain identities, it is feasible to generate particular brain regions of interest by applying novel modified protocols for guiding and directing regional identity (Kadoshima et al., 2013; Pasca et al., 2015; Tanaka et al., 2020). Brain organoids recapitulate many features of the fetal human brain, including cytoarchitecture, cell diversity and maturation and comprise a variety of cell types comparable, to some extent, to the complex composition of the cells present in the brain (reviewed in Chiaradia and Lancaster, 2020). Importantly, spontaneous neuronal activity has been detected in brain organoids suggesting the existence of functional communication among neuronal cells (Lancaster et al., 2013). Brain organoids have been used for modeling neurological diseases and NDDs, providing remarkable advantage in studying diseases *in vitro*, in a 3D environment resembling the affected tissue (reviewed in Chiaradia and Lancaster, 2020). The position of organoids at the interface of *in vitro* and *in vivo* neurobiology makes them a unique model system that will provide further progress in understanding brain development (Chiaradia and Lancaster, 2020). Combined with single cell transcriptomics technology, the brain organoids would enable to decipher cellular heterogeneity and transcriptional landscape at single cell resolution. These novel tools will open innovative approaches for studying the roles of SOX TFs in brain development at the single cell level in physiological and pathological conditions.

In the past decade, the “omics” technologies, such as genomics, transcriptomics, miRNomics, and proteomics have become integrated parts of the research in biology and medicine, enabling progress in collecting, processing and integrating huge amounts of health-related information (D’Adamo et al., 2020). While genomic analyses provide the insight into variation at DNA level, RNAseq data reveal transcriptome diversity in patients compared to healthy controls. For instance, integrative transcriptomic analysis may lead to the identification of key deregulated candidate genes and pathways shared between various developmental disorders. Such innovative approach may help in identifying novel roles of SOX proteins in pathology of NDDs.

Advance in proteomic technologies enables mapping of specific gene interactome providing the insights into the network of interacting partners. Thus, the study of Nanog interactome identified SOX2 as interacting factor (Gagliardi et al., 2013). Mapping the interactome of specific SOX protein will provide deeper insight into interacting factors and disruption of interactions in diverse pathological conditions. Appropriate bioinformatics analysis will reveal networks of TFs and signaling pathways differentially regulated between different cell states. Such analysis will identified position of SOX proteins within signaling cascades active in particular cell context and pinpoint their functionally relevant links in complex regulatory networks.

Although many important roles during neural development have been assigned to SOX TFs, we strongly believe that many novel functions are yet to be discovered.

## Author Contributions

All authors wrote the manuscript and contributed to literature collection. MiS designed the concept of the manuscript and supervised and edited the manuscript. MiS, DD, and DSN contributed to the preparation of the tables. MaS, AL, MM, and DSN designed the figures. All authors contributed to the article and approved the submitted version.

## Conflict of Interest

The authors declare that the research was conducted in the absence of any commercial or financial relationships that could be construed as a potential conflict of interest.

## Abbreviations

TFs: transcription factors
ESCs: embryonic stem cells
RA: retinoic acid
iPSCs: induced pluripotent stem cells
NPCs: neural progenitor cells
CNS: central nervous system
NSCs: neural stem cells
NDDs: neurodevelopmental disorders
SVZ: subventricular zone
SGZ: subgranular zone
TCF/LEF: T cell factor/Lymphoid enhancer factor
RARs: retinoic acid receptors
RXRs: retinoid X receptors
RAREs: *cis*-acting RA response elements
CRMs: *cis*-regulatory modules
miRNAs: microRNAs.
