# Supplementary material for: SOX Transcription Factors as Important Regulators of Neuronal and Glial Differentiation During Nervous System Development and Adult Neurogenesis
Source: Front Mol Neurosci. 2021 Mar 31;14:654031. doi: 10.3389/fnmol.2021.654031 (PMC8044450; doi:10.3389/fnmol.2021.654031)
Supplement: Supplementary file 1 [file Table_1.DOCX]

Supplementary Material

**Supplementary Table S1:** *SOX* gene variants associated with NDDs

| **Genetic Aetiology** | **Clinical presentation** | **References** |
| --- | --- | --- |
| ***SOX3* deletion**  Xq26.2-27.2 deletion (comprising the *Factor IX* gene and *S0X3*) | intelectual disability, haemophilia B | ([Stevanovic et al., 1993](#_ENREF_202)) |
| ***SOX3* deletion**  2.1 Mb Xq27.1-q27.2 deletion (comprising the entire *SOX3* gene) | mild intellectual disability, language delay, dysarthria, behavior problems, minor facial anomalies, hyperphagia | ([Helle et al., 2013](#_ENREF_75)) |
| ***SOX3* duplication**  Xq26.3-27.3 duplication | intellectual disability, growth hormone deficiency, ocular dyspraxia | ([Stagi et al., 2014](#_ENREF_195)) |
| ***SOX3* duplication**  323.8 kb Xq27.1 duplication  (breakpoints 139,261,842–139,585,653) | severe intellectual disability, hypoglycemia, prolonged jaundice, failure to thrive, micropenis, small-volume testes, adrenal insufficiency, central hypothyroidism, hypoplastic anterior pituitary, growth hormone deficiency, ventricular septal defect, patent ductus arteriosus, trivial mitral regurgitation | ([Arya et al., 2019](#_ENREF_9)) |
| ***SOX3* duplication**  396 kb Xq27.1 duplication (breakpoints 139,347,578–139,743,254) | severe intellectual disability, neuropathic bladder, lumbar myelomeningocele, hydrocephalus, agenesis of the corpus callosum, hypoglycaemia, micropenis, small-volume testes, growth hormone deficiency, bilateral optic atrophy, left temporal lobe epilepsy, Arnold-Chiari malformation, limited mobility | ([Arya et al., 2019](#_ENREF_9)) |
| ***SOX3* duplication**  11 Mb Xq27.1 duplication (breakpoints 139,055,504–150,083,888) | mild intellectual disability, short stature, growth hormone deficiency, borderline TSH deficiency, hypoplastic anterior pituitary | ([Arya et al., 2019](#_ENREF_9)) |
| ***SOX3* duplication**  481 kb Xq27.1 duplication  (breakpoints 139,261,841–139,743,254) | moderate intellectual disability, short stature, pubertal delay, low testicular volumes, moderate learning difficulties, growth hormone deficiency, partial agenesis of the corpus callosum, absent septum pellucidum, presence of heterotopic grey matter | ([Arya et al., 2019](#_ENREF_9)) |
| ***SOX3***  missense variant c.449C>A (p.Ser150Tyr) | mild intellectual disability, microphthalmia, coloboma, hypopituitarism, facial dysmorphology, dental anomalies, microcephaly, retrognathia, solitary median maxillary central incisor | ([Jelsig et al., 2018](#_ENREF_87)) |
| ***SOX3***  in-frame duplication of 33 bp encoding for 11 alanines in a polyalanine tract of the *SOX3* gene | intellectual disability, growth hormone deficiency | ([Laumonnier et al., 2002](#_ENREF_113)) |
| ***SOX4***  heterozygous missense variants:  c.198C>A (p.Phe66Leu); c.334G>C (p.Ala112Pro); c.176T>G (p.Ile59Ser);  c.315G>T (p.Lys105Asn) | common features: developmental delay, intellectual disability, mild facial and digital morphological abnormalities | ([Zawerton et al., 2019](#_ENREF_251)) |
| ***- SOX5*** intragenic heterozygous deletions ranging from 72 kb to 466 kb;  - balanced *de novo* translocation with breakpoint within ***SOX5*** [46,XX,t(11;12)(p13;p12.1)dn];  ***- SOX5*** deletions  12p12 deletions ranging from 1.4 Mb to 12.1 Mb, encompassing multiple genes including *SOX5* | common features: intellectual disability, prominent language delay, behavior abnormalities, dysmorphic appearance | ([Lamb et al., 2012](#_ENREF_110)) |
| ***SOX5*** deletion  heterozygous 12p12.1 deletions ranging from 120 kb to 4.9 Mb | common features: intellectual disability, moderate delay in motor development, delayed speech development | ([Schanze et al., 2013](#_ENREF_186)) |
| ***SOX5***  heterozygous stop gain variant in exon eight  (c.1021G>T, p. (G341*)) | intellectual disability, moderate developmental delay, bilateral optic atrophy, mildly dysmorphic features, scoliosis, behavioral issues | ([Nesbitt et al., 2015](#_ENREF_144)) |
| ***SOX6***  heterozygous variants – CNVs (partial deletions of *SOX6* which did not involve any other gene), SNVs (nonsense, frameshift, missense variants), balanced reciprocal translocation 46,XY,t(2;11)(p11.2;p15.2) | common features: intellectual disability, developmental delay  inconstant features: attention-deficit/hyperactivity disorder, autism, mild facial dysmorphism, craniosynostosis, multiple osteochondromas | ([Tolchin et al., 2020](#_ENREF_218)) |
| ***SOX11***  heterozygous missense mutations localize within the HMG domain:  c.347A>G (p.Tyr116Cys); c.178T>C (p.Ser60Pro) | common features: mild intellectual disability, dysmorphic facial features, microcephaly, growth deficiency, hypoplastic fifth toe nails | ([Tsurusaki et al., 2014](#_ENREF_222)) |
